# Salivary CD5L as a Potential Non-Invasive Biomarker for Pathological Staging and Prognostic Assessment in Oral Squamous Cell Carcinoma

**DOI:** 10.3390/diagnostics16121856

**Published:** 2026-06-16

**Authors:** Nan-Chin Lin, Yu-Hsin Tseng, Novaria Sari Dewi Panjaitan, Kuo-Yang Tsai, Kuan-Min Huang, Wan-Chen Lan, Tzong-Ming Shieh, Yin-Hwa Shih

**Affiliations:** 1 Department of Oral and Maxillofacial Surgery, Show Chwan Memorial Hospital, Changhua 500, Taiwan; tifa92180@gmail.com (N.-C.L.); tsaikuoyang@gmail.com (K.-Y.T.); sonning919@gmail.com (K.-M.H.); 2 Department of Pediatrics, Kaohsiung Medical University Hospital, Kaohsiung Medical University, Kaohsiung 807378, Taiwan; grapepuff@gmail.com; 3 Center for Biomedical Research, Research Organization for Health, National Research and Innovation Agency (BRIN), Cibinong 10340, Indonesia; nova014@brin.go.id; 4 School of Dentistry, China Medical University, Taichung 404328, Taiwan; magic1986713@hotmail.com; 5 Department of Healthcare Administration, Asia University, Taichung 413305, Taiwan

**Keywords:** salivary biomarker, CD5L, late-stage OSCC, non-invasive diagnosis, pathological staging

## Abstract

**Background/Objectives**: In Taiwan, more than 50% of patients with oral cancer seek medical help at a late stage. Reliable non-invasive biomarkers for pathological staging and disease monitoring are still lacking. This study aimed to identify a specific biomarker associated with late-stage oral cancer and to develop a non-invasive strategy for early pathological diagnosis and disease monitoring with improved patient acceptability. **Methods**: A total of 116 participants were enrolled, including 31 patients with early-stage oral cancer, 49 with late-stage oral cancer, and 36 healthy controls. Saliva samples were collected for proteomic analysis, and the findings were validated using ELISA and tissue immunohistochemistry. The identified biomarker was validated, and its tumor-promoting role was confirmed using malignant phenotype assays, including colony formation, soft agar, migration, and invasion assays. **Results**: Our results demonstrate that CD5L expression could not be distinguished between early- and late-stage groups using tissue immunohistochemistry. In contrast, salivary CD5L levels differentiated early-stage from late-stage patients noninvasively. Functional studies demonstrated that CD5L suppression markedly attenuated malignant phenotypes (colony formation, anchorage-independent growth, migration, invasion), suggesting its involvement in tumor aggressiveness and metastatic potential. **Conclusions**: These findings provide new insights into the pathological role of salivary CD5L in oral cancer progression and support its potential as a non-invasive biomarker for disease stratification.

## 1. Introduction

According to the 2018 Taiwan Cancer Registry and the 2020 Cause of Death Statistics released by the Ministry of Health and Welfare, more than 3000 people die from oral cancer each year in Taiwan, and over 8000 individuals are newly diagnosed annually [1]. The 5-year disease-free survival rate of patients with oral cancer decreased approximately 40% from early stage to late stage [2]. Hence, the Taiwan government provides free oral health examination to individuals with these high-risk behaviors (cigarette smoking, alcohol consumption, and betel quid chewing) to reduce cancer mortality and improve prognosis [3]. Although the importance and benefits of early screening and treatment are well recognized, more than 50% of patients in Taiwan still delay seeking medical help in the advanced stage [4]. Therefore, investigating specific biomarkers in patients at advanced stages remains crucial [5,6].

The clinical diagnosis of oral cancer primarily relies on outpatient examination, during which physicians perform visual inspection and palpation of the oral mucosa, maxilla and mandible, tongue, and cervical lymph nodes. In addition, factors such as the patient’s age, past medical history, family history, and exposure to known risk factors are evaluated to determine whether the individual belongs to a high-risk population for oral cancer. If clinicians manage oral lesions solely with pharmacological treatment without further pathological evaluation, such as biopsy, patients with oral cancer may experience delayed diagnosis and treatment [7]. Given the heterogeneous clinical presentation of oral cancer, biopsy-based histopathological examination remains essential for establishing an accurate diagnosis and determining disease stage. However, many patients are reluctant to undergo biopsy examination at the early stage of lesion development [8]. Because invasive diagnostic procedures may provoke fear and lead to patient refusal, numerous non-invasive approaches for early-stage oral cancer screening and detection have been developed in recent years [9,10].

Biomolecules in body fluids are commonly used as disease biomarkers [11,12], and the non-invasive collection of saliva makes them highly acceptable to the general population [13,14]. In recent years, numerous studies have demonstrated that saliva contains abundant molecules [15,16] that could serve as biomarker-rich substances for medical applications. Saliva-derived DNA is widely applied in forensic genotyping [17], while RNA has been used for viral infection detection [18]. Immunoglobulins present in saliva are used to diagnose human immunodeficiency virus and hepatitis virus infections [19]. In addition, drug residues in saliva are used to monitor substance abuse and drug metabolism [20]. Saliva also enables highly accurate detection of oral diseases. Biomarkers such as interleukins, growth factors, and enzymes have been associated with clinical outcomes, including recurrence, disease progression, and prognosis [21,22,23,24,25].

Despite advances in diagnostic approaches, reliable non-invasive biomarkers for distinguishing pathological stages of oral cancer remain limited. This study is expected to have a significant societal impact by advancing a non-invasive, cost-effective, and patient-friendly strategy for the early detection and disease stratification of oral cancer. Ultimately, the findings may alleviate healthcare burden associated with advanced disease and reduce cancer mortality.

## 2. Materials and Methods

### 2.1. Study Design

The cross-sectional study was conducted using a convenience sampling method. Eligible patients were screened according to predefined inclusion criteria. After obtaining informed consent, saliva samples were collected from all participants, and tumor tissue was obtained from patients with OSCC (oral squamous cell carcinoma) during surgery. Saliva samples from different groups were subjected to proteomics analysis to identify candidate biomarkers associated with late-stage OSCC. The identified biomarker was subsequently validated using enzyme-linked immunosorbent assay (ELISA) and tissue immunohistochemistry (IHC). Functional assays were performed to evaluate the biomarker’s role in malignant phenotypes. Specifically, loss-of-function experiments using siRNA-mediated knockdown were conducted to assess its effects on tumor cell behavior.

### 2.2. Ethical Approval

This study was conducted following approval from the Institutional Review Board (IRB) of Show Chwan Memorial Hospital (Approval No. 1130701 during 1 November 2024 to 31 October 2025). All procedures involving human participants were performed in accordance with the ethical standards of the institutional research committee and the principles of the Declaration of Helsinki. Written informed consent was obtained from all participants prior to enrollment.

### 2.3. Study Population and Eligibility Criteria

Participants were recruited and categorized into three groups: early-stage oral cancer (*n* = 31), late-stage oral cancer (*n* = 49), and healthy controls with no lesions (*n* = 38). Inclusion criteria were as follows: (1) patients with histopathologically confirmed oral cancer who could be classified as early-stage or late-stage according to pathological staging, and (2) lesion-free healthy controls without clinically evident oral lesions. For all participants, sex, age, lesion site, treatment modality, pathological stage, and habits of smoking, betel nut chewing, and alcohol consumption were recorded. The early stage refers to TNM stages 0, I, and II, and the late stage refers toTNM stages III and IV.

### 2.4. Saliva and Tissue Collection

Due to numerous confounding factors related to patients’ health status, we conducted a proteomic analysis of healthy controls, early-stage oral cancer patients, and late-stage oral cancer patients without specifically excluding individuals with chronic diseases. Participants were provided with a saliva collection kit (Salivette^®^, SARSTEDT, Nümbrecht, Germany). The saliva was collected before surgery and treatment. An absorbent cotton swab was placed on the tongue for 5 min to collect 1–2 mL of whole saliva, after which the swab was returned to the collection tube. The collection tube (~2 mL saliva) was centrifuged at 1000× *g* for 5 min. The supernatant was centrifuged at 2000× *g* for 5 min to remove oral epithelial cell debris and obtain cell-free saliva.

### 2.5. Proteomics Analysis

We randomly selected five samples from each collected specimen group. Cell-free saliva samples were lysed using RIPA buffer (Millipore, Burlington, MA, USA), and protein concentration was determined using a bicinchoninic acid (BCA) assay. Protein samples (5 μg) were separated by SDS–PAGE until the dye front reached the boundary between the stacking and resolving gels. The protein-containing gel was cut down and subjected to in-gel digestion. The procedures for in-gel digestion and LC–MS/MS analysis were performed as described in our previously published study [26]. Differential expression analysis was performed using Student’s *t*-test (*n* = 5 in each group). *p*-values were adjusted using the Benjamini–Hochberg false discovery rate (FDR). Proteins with FDR < 0.05 and fold change > 1.5 were considered significantly differentially expressed.

### 2.6. Enzyme-Linked Immunosorbent Assay (ELISA)

Salivary CD5L levels were quantified using a commercially available human CD5L ELISA kit (Invitrogen, Carlsbad, CA, USA, Cat. No. EH94RB) according to the manufacturer’s instructions. Briefly, 100 μL of standards and appropriately diluted samples were added to each well of a pre-coated microplate and incubated overnight at 4 °C with gentle shaking (40 rpm). Following incubation, the wells were aspirated and washed four times with 1× wash buffer. Subsequently, 100 μL of diluted biotin-conjugated antibody was added to each well, and the plate was incubated for 1 h at room temperature with gentle shaking (40 rpm). After washing the wells four times, 100 μL of streptavidin–horseradish peroxidase (HRP) solution was added and incubated for 45 min at room temperature with gentle shaking (40 rpm). The wells were then washed four times, followed by the addition of 100 μL of tetramethylbenzidine (TMB) substrate solution. The plate was incubated for 30 min at room temperature in the dark. The reaction was terminated by adding 50 μL of stop solution to each well, and absorbance was immediately measured at 450 nm using a microplate reader. The concentration of CD5L in each sample was calculated by interpolation from the standard curve using a four-parameter logistic (4PL) regression model. The calculated concentrations were further adjusted according to the sample dilution factor. Final results were expressed as pg/mL.

### 2.7. Immunohistochemical Staining Quantification

Immunohistochemical staining was quantitatively analyzed using Fiji (ImageJ 1.54p) software [27]. Representative images were captured under identical microscope settings. Regions of interest (ROIs) containing viable tumor tissue were selected, while necrotic, keratinized, and artifact-prone areas were excluded from analysis. Color deconvolution was performed using the H DAB setting to separate hematoxylin and DAB signals. The DAB channel was converted to 8-bit grayscale, and a uniform threshold was applied to all images to identify positive staining areas. The percentage of DAB-positive area (area fraction), mean gray value, and integrated density were measured. At least three representative fields per sample were analyzed, and the mean value was used for statistical analysis. Mean intensity represents the average staining intensity within the selected region of interest, whereas integrated intensity reflects the cumulative staining signal incorporating both staining intensity and positively stained area.

### 2.8. Cell Culture and Arecoline Treatment

Gingival carcinoma neck metastasis (GNM) cells were obtained and authenticated by Professor Cheng-Chia Yu of Chung Shan Medical University. GNM was maintained in DMEM (GIBCO #12100-046) supplemented with 10% fetal bovine serum (Corning 35-010-CV) and 1% penicillin–streptomycin (GIBCO #15240-062) at 37 °C in a humidified incubator with 5% CO_2_. For arecoline treatment, cells were exposed to arecoline (Sigma-Aldrich, St. Louis, MO, USA) at a final concentration of 50 μg/mL for 24 h prior to protein extraction.

### 2.9. Western Blotting

Total protein was quantified using a bicinchoninic acid (BCA) assay. Equal amounts of protein were then separated by SDS–PAGE using a Bis–Tris gel system and transferred to PVDF membranes for Western blot analysis. Immunoreactive signals were visualized and captured using a chemiluminescence imaging system (Fusion SOLO, Vilber Lourmat, Collégien, France). The primary and secondary antibodies used in this study and their respective dilution ratios were as follows: CD5L (Thermo Fisher Scientific, Waltham, MA, USA, #PA5-84779, 1:1000); GAPDH (Proteintech, Rosemont, IL, USA, #60004-1-Ig, 1:10,000); goat anti-mouse IgG secondary antibody (Jackson ImmunoResearch, West Grove, PA, USA, #115-035-003, 1:10,000); and goat anti-rabbit IgG secondary antibody (Jackson ImmunoResearch, #111-035-003, 1:10,000).

### 2.10. CD5L Knockdown

GNM cells were seeded in 6-well plates and cultured to approximately 60% confluence for 24 h, with 3 replicates per group. siRNA, Lipofectamine^®^ RNAiMAX Reagent (Thermo Fisher Scientific, Waltham, MA, USA), and Opti-MEM^®^ Medium (Gibco, Thermo Fisher Scientific, Waltham, MA, USA) were thoroughly mixed and incubated at room temperature for 5 min before being added to the cells. The cells were then returned to the incubator, and one plate of cells was harvested each day for CD5L protein analysis. The siRNA kit was purchased from Eurogentec and included three siRNA sequences.

### 2.11. Population Doubling Time Calculation

Twenty-four hours after siRNA transfection, 4000 cells were seeded into 48-well plates. Cells were counted every 24 h for five consecutive days. Cell numbers from days 3 to 5 were used to calculate the population doubling time using an online calculator (Żuławińska, J. Cell Doubling Time Calculator. Available at: https://www.omnicalculator.com/biology/cell-doubling-time. Accessed: 30 December 2025).

### 2.12. Soft Agar Assay

This experiment evaluated the ability of cells to grow under anchorage-independent (suspension) conditions, which is a hallmark of malignant phenotypes. A 24-well plate was prepared with a bottom layer of DMEM supplemented with FBS containing 1% agar (Neogen, Lansing, MI, USA). Cells were then mixed with DMEM supplemented with FBS containing 0.7% agarose (Thermo Fisher Scientific, Waltham, MA, USA) and seeded as the top layer. Five thousand cells were seeded per well. One milliliter of culture medium was added weekly. Each group was tested in triplicate. After two weeks, colonies were stained with 0.005% crystal violet for 1 h, washed to remove excess solution, and air-dried. Colony numbers and area were quantified using ImageJ software (National Institutes of Health, Bethesda, MD, USA).

### 2.13. Colony Formation Assay

For the colony formation assay, 2000 cells were seeded per well in 6-well plates. After 1 week, cells were fixed with methanol: acetic acid (3:1) for 10 min and stained with 0.5% crystal violet for 10 min. Excess stain was removed by washing with ddH_2_O, and the plates were air-dried. Colonies were observed under a microscope, and a colony was defined as a cluster containing more than 32 cells. Each group was analyzed in triplicate. For quantification, five random fields per well were photographed, and the average colony count was calculated.

### 2.14. Migration Assay

This experiment evaluated cell migratory ability, as more malignant cells typically migrate faster. A total of 40,000 cells were seeded per well in 6-well plates and cultured for 24 h. A straight scratch was created across the center of each well using a 200 μL pipette tip held perpendicular to the plate. The wells were washed three times with PBS to remove detached cells, and fresh culture medium was added. Images were captured at 8, 12, 24, 48, and 72 h. Wound closure was quantified using ImageJ (National Institutes of Health, Bethesda, MD, USA) via pixel analysis to determine the wound-healing rate. Each group was tested in triplicate.

### 2.15. Invasion Assay

This experiment evaluated cells’ ability to penetrate a collagen-coated matrix layer; greater penetration capacity indicates greater invasive potential. A commercial invasion assay kit (Abcam, Cambridge, UK) was used according to the manufacturer’s instructions. Cells transfected with siRNA were subjected to serum starvation for 24 h prior to the assay. A 96-well plate was prepared by adding 150 μL of culture medium containing 10% FBS to the lower chamber. A total of 50,000 suspended cells in 100 μL were seeded into the upper chamber of a collagen-coated transwell insert. The insert was then placed into the 96-well plate and incubated for 48 h. After incubation, non-invading cells on the upper surface of the transwell membrane were gently removed with a cotton swab. The medium in the lower chamber was aspirated, and the wells were washed with PBS. Then, the cell staining solution provided in the kit was added. The transwell insert was returned to the 96-well plate and incubated at 37 °C for 1 h for staining. During the incubation period, a standard curve for cell number was prepared. After staining for 1 h, fluorescence intensity was measured using a microplate reader with excitation/emission settings of 530/590 nm. The measured fluorescence values were then interpolated from the standard curve to determine the number of invaded cells. The fluorescence reader we used was a Varioskan™ LUX multimode microplate reader (Thermo Fisher Scientific, Waltham, MA, USA).

### 2.16. Statistics Analysis

All statistical analyses were performed using SPSS (version 25; IBM Corp., Armonk, NY, USA). Demographic and clinicopathological characteristics were summarized using descriptive statistics. The correlations between demographic or pathological variables and CD5L expression levels were evaluated using Spearman’s rank correlation analysis. Differences among groups were assessed using Welch’s analysis of variance (ANOVA) due to unequal variances among groups, as indicated by Levene’s test. Post hoc pairwise comparisons were performed using the Games–Howell test, which does not assume equal variances or equal sample sizes. Differences in malignant phenotypes following CD5L knockdown between the two groups were analyzed using independent-samples *t*-tests. A *p*-value < 0.05 was considered statistically significant.

## 3. Result

### 3.1. Subsection Characteristics of Participants

A total of 116 participants were included in this study, comprising 36 healthy controls, 31 patients with early-stage OSCC, and 49 patients with late-stage OSCC. Most participants were male across all groups. The average age of the healthy controlS (48.47 ± 18.01) is lower than that of early-stage (58.89 ± 10.68) and late-stage (57.74 ± 9.99) patients (*p* < 0.05). Significant differences in lifestyle risk factors were observed among the groups. The prevalence of alcohol consumption, betel nut chewing, and cigarette smoking was significantly higher in patients with OSCC compared with healthy controls (all *p* < 0.001). The data indicate that a high proportion of patients with oral cancer have a history of areca nut chewing, cigarette smoking, and alcohol consumption. In the OSCC group, the majority of patients presented with primary cancer (primary and second primary), where 13~14% had recurrent cancer and 3~4% had metastatic cancer. No significant difference in cancer status was observed between the early- and late-stage OSCC groups (*p* = 0.513, Cramer’s V = 0.171). More than 80% of cases in both the early- and late-stage groups involved tumors located in the buccal mucosa and gingiva (Table 1).

### 3.2. Proteomics Analysis Showed CD5L Was the Candidate Biomarker of Late-Stage OSCC

**Figure 1:**
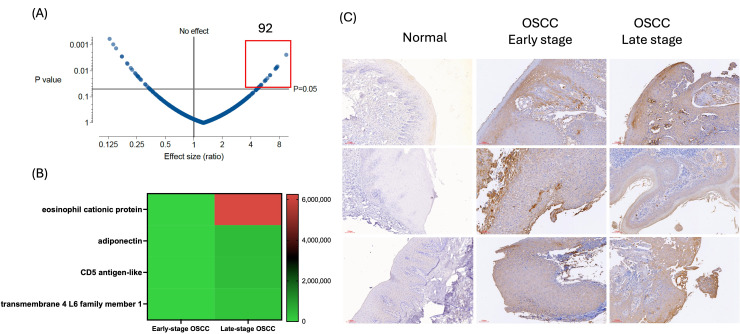
(**A**) CD5L is the candidate biomarker expressed in late-stage OSCC. Volcano plot showing proteins significantly upregulated (>3-fold, *p* < 0.05) in OSCC (*n* = 5 in each group). A total of 92 candidate proteins were identified. (**B**) Heatmap visualization of biomarker expression levels among study groups. The heatmap illustrates the relative expression levels of selected biomarkers across the study groups. Color intensity represents normalized expression levels, ranging from low expression (green) to high expression (red). Four proteins, including eosinophil cationic protein, adiponectin, CD5 antigen-like, and transmembrane 4 L6 family member 1 expression, were relatively higher in late-stage OSCC than in early-stage OSCC. (**C**) Immunohistochemical staining showed elevated CD5L expression in OSCC tumor tissues, with stronger staining in tumors. Dark brown staining in the figure indicates CD5L expression. *n* = 3 in each group. Scale bar = 100 μm.

To identify potential salivary biomarkers associated with OSCC, saliva samples from healthy controls (*n* = 5), early-stage OSCC patients (*n* = 5), and late-stage OSCC patients (*n* = 5) were subjected to proteomic analysis. A total of 92 proteins showing significantly increased expression in OSCC (>3-fold, *p* < 0.05) were identified (Figure 1A). From the 92 candidate proteins identified in the proteomic analysis, proteins showing relatively lower expression in both the healthy control and early-stage oral cancer groups but higher expression in the late-stage oral cancer group were selected. Following this filtering step, four candidate proteins remained for further evaluation (Figure 1B). Among these candidates, CD5L demonstrated consistently elevated expression in ELISA (Table 2), particularly in late-stage OSCC, and was therefore selected for further Immunohistochemical (IHC) validation (Table 3 and Figure 1C).

Salivary CD5L levels revealed substantial variability across the three groups. The late-stage group exhibited the highest mean value (345.96 ± 303.54), followed by the early-stage group (181.44 ± 124.27) and the healthy control group (173.94 ± 91.82). No significant difference in CD5L levels was observed between the healthy control group and the early-stage OSCC group (mean difference = 42.81, *p* = 0.48). However, salivary CD5L levels were significantly higher in the late-stage OSCC group compared with the healthy control group (mean difference = 121.70, *p* = 0.02, Cohen’s d = 0.77) and the early-stage OSCC group (mean difference = 164.51, *p* < 0.001, Cohen’s d = 0.7) (Table 2).

**Figure 2:**
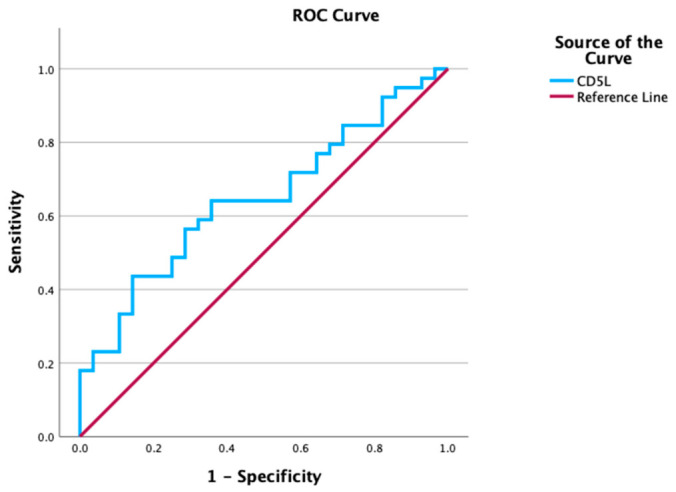
ROC curve of CD5L for distinguishing early-stage from late-stage oral cancer. The AUC was 0.647 (95% CI: 0.515–0.778, *p* = 0.029).

ROC curve analysis demonstrated that salivary CD5L significantly discriminated early-stage from late-stage oral cancer, with an AUC of 0.647 (95% CI: 0.515–0.778, *p* = 0.029), indicating modest but statistically significant discriminative ability (Figure 2).

IHC analysis further demonstrated that CD5L expression was elevated in OSCC tumor tissues, with stronger staining in both early-stage and late-stage tumors (Figure 1C). We quantified the CD5L signal using ImageJ and calculated the difference among groups. Data revealed that both early- and late-stage groups exhibited significantly lower mean intensity but higher integrated density and area fraction than the healthy control group (all *p* < 0.001, Welch ANOVA). For integrated density, both early-stage (63,835,296 ± 32,366,842) and late-stage groups (72,058,214 ± 33,487,621) exhibited markedly higher values compared to healthy controls (12,601,174 ± 11,079,623) (both *p* < 0.001 with effect size Cohen’s d ≈ 2). However, there was no statistically significant difference between early- and late-stage groups (*p* = 0.42). Similarly, area fraction (%) was significantly higher in the early-stage (48.33 ± 20.58) and late-stage (55.88 ± 20.42) groups than in healthy controls (8.98 ± 7.33) (both *p* < 0.001 with effect size Cohen’s d > 2). No significant difference was found between early- and late-stage groups (*p* = 0.27) (Table 3).

These findings suggest that salivary CD5L, as a non-invasive biomarker, has the potential to distinguish between early- and late-stage oral cancer. Compared with immunohistochemical staining, which requires invasive tissue sampling, salivary detection offers a more convenient and rapid alternative.

### 3.3. CD5L Expression Was Elevated in Oral Squamous Cell Carcinoma Cell Lines and Was Upregulated by Arecoline

**Figure 3:**
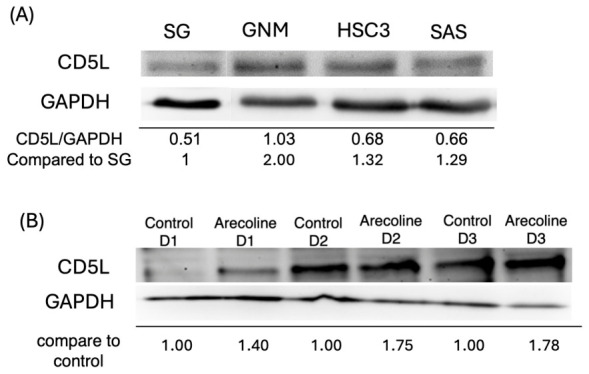
CD5L expression in oral cell lines (**A**) Comparison of CD5L expression among oral cell lines. SG represents a non-OSCC cell line, while GNM, HSC-3, and SAS are OSCC cell lines. CD5L expression followed the order: GNM > HSC-3 > SAS > SG. (**B**) Arecoline treatment for 3 days (D1 to D3) significantly increased CD5L expression in GNM cells. Control represents vehicle-treated (DMSO) control cells.

To investigate the function of CD5L expression in oral cells, we examined CD5L expression in several oral squamous cell carcinoma (OSCC) cell lines available in our laboratory and compared them with SG cells (human gingival epithelial cells) as a healthy control. The OSCC cell lines, including GNM (gingival-derived oral squamous cell carcinoma), HSC-3 (human oral squamous cell carcinoma-3), and SAS (human tongue squamous cell carcinoma), exhibited higher CD5L expression than the non-OSCC SG cells. Among the OSCC cell lines, CD5L expression was highest in GNM cells, followed by HSC-3 and SAS cells. Furthermore, treatment of GNM cells with arecoline resulted in an increased trend in CD5L expression. These findings suggest that exposure to arecoline, a major alkaloid of areca nut, may upregulate CD5L expression in oral cancer cells, indicating a potential link between betel nut chewing and increased CD5L expression in OSCC patients (Figure 3).

### 3.4. RNAi Knockdown of CD5L Reduces the Malignant Phenotype of GNM Cells

**Figure 4:**
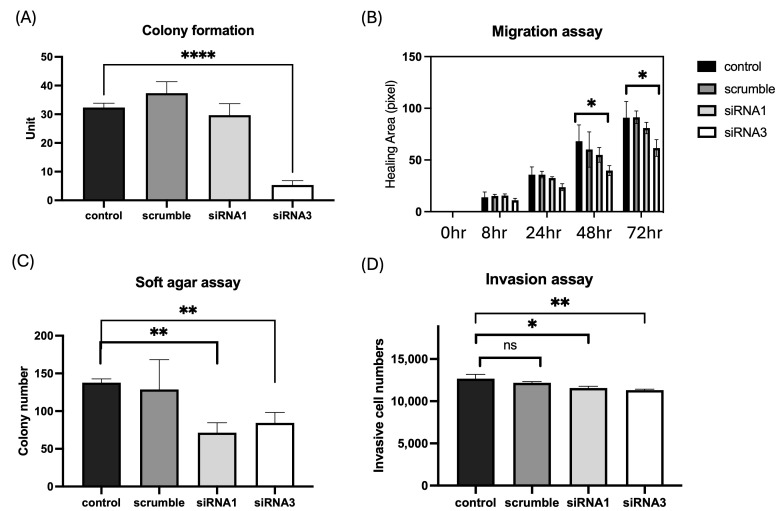
Effects of CD5L knockdown on malignant phenotypes of GNM cells. (**A**) Colony formation assay. (**B**) Migration assay. (**C**) Soft agar assay. (**D**) Invasion assay. Experiments were performed in three independent experiments. Data are presented as mean ± SD. Statistical significance was determined using Student’s *t*-test. * *p* < 0.05, ** *p* < 0.01, **** *p* < 0.0001.

**Figure 5:**
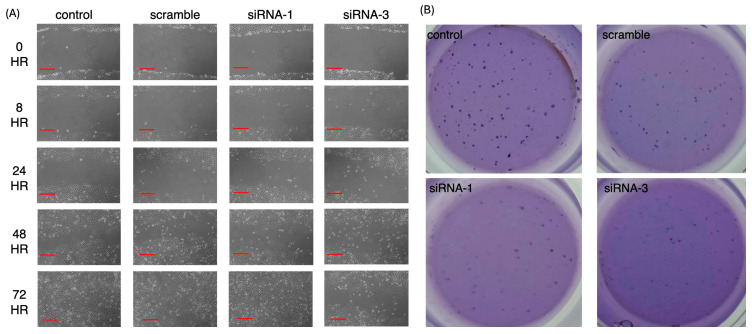
Representative images of migration and colony formation assays following CD5L knockdown in GNM cells. (**A**) Migration assay images of GNM cells in different groups at 0, 8, 24, 48, and 72 h. Scale bar = 250 μm. (**B**) Colony formation assay images of GNM cells in different groups.

Commercial siRNAs were used to knock down CD5L expression in GNM cells. Among the tested siRNAs, siRNA-1 and siRNA-3 showed the most effective suppression of CD5L expression (Supplementary Figure S1A). We further evaluated the effect of CD5L knockdown on cell proliferation by measuring the population doubling time. Suppression of CD5L expression by siRNA-1 and siRNA-3 did not reduce the population doubling time of GNM cells compared with the control group (Supplementary Figure S1B). To determine whether CD5L contributes to the malignant behavior of OSCC cells, CD5L expression was silenced in GNM cells using siRNA. Knockdown of CD5L significantly reduced colony formation ability in the siRNA-3 group (Figure 4A and Figure 5B). In the migration assay, CD5L suppression significantly decreased the migration rate of GNM cells compared with the control group (Figure 4B and Figure 5A). The soft agar assay further demonstrated a reduction in anchorage-independent colony formation (Figure 4C). Consistently, the invasion assay showed a decreased invasive capacity following CD5L knockdown (Figure 4D). These findings indicate that CD5L promotes malignant phenotypes in OSCC cells.

## 4. Discussion

In the present study, baseline characteristics indicated that patients with oral cancer were predominantly male and exhibited a higher prevalence of risk behaviors (A, B, and C). Tumors were mainly located in the buccal mucosa and gingiva, consistent with previously reported epidemiological patterns of oral cancer in Taiwan [13,28,29]. We identified CD5L as a potential biomarker associated with advanced OSCC using salivary proteomic analysis. Subsequently, salivary and tissue CD5L expression was further validated by ELISA and immunohistochemistry. Our results demonstrated that CD5L expression was increased in OSCC tissues and saliva, particularly in the saliva of patients with late-stage disease. Salivary CD5L expression more clearly discriminates between early- and late-stage oral cancer and, compared with IHC staining, enables non-invasive differentiation at the initial time point. This feature has important clinical implications for screening and underscores its potential utility as a non-invasive biomarker for disease staging.

In addition, CD5L expression was higher in OSCC cell lines compared with non-OSCC epithelial cells. Functional studies further showed that CD5L knockdown reduced colony formation, migration, invasion, and anchorage-independent growth in GNM cells, suggesting that CD5L contributes to the malignant phenotype of OSCC cells.

CD5L, also known as apoptosis inhibitor of macrophage (AIM), is a secreted protein belonging to the scavenger receptor cysteine-rich (SRCR) superfamily. CD5L plays important roles in immune regulation, lipid metabolism, and inflammatory responses. It has also been implicated in tumor progression and in regulating the tumor microenvironment by modulating inflammation surrounding cancer cells [30]. CD5L has been identified as a potential biomarker in lung adenocarcinoma, lung squamous cell carcinoma, and hepatocellular carcinoma tissues [31,32,33]. Elevated vascular CD5L expression in cancer patients has been associated with resistance to bevacizumab and poorer overall survival [34]. Taken together, the evidence from the literature shows that CD5L has significant clinical utility as a diagnostic and prognostic biomarker. This study intentionally did not exclude patients with chronic diseases or potential inflammatory conditions, thereby more closely reflecting real-world clinical populations. Despite possible immune and inflammatory confounders, CD5L remained significantly elevated in late-stage OSCC and demonstrated the ability to discriminate between early- and late-stage disease. These findings suggest that CD5L may retain potential clinical utility under heterogeneous inflammatory conditions, although further validation is required.

Although histopathological staging remains the gold standard for OSCC diagnosis and staging, saliva-based biomarkers may still provide complementary, non-invasive information on tumor biology. The present study did not directly evaluate occult tumors or metastatic disease; therefore, the utility of CD5L for metastasis detection remains unclear. Nevertheless, the observed elevation of CD5L in late-stage OSCC, together with its association with malignant phenotypes in cell-based experiments, suggests that CD5L may reflect tumor aggressiveness and disease progression rather than serving solely as a staging marker.

Our loss-of-function experiments further demonstrated that CD5L knockdown significantly reduced several malignant phenotypes in OSCC cells, including colony formation, migration, and invasion. These findings suggest that CD5L may promote tumor aggressiveness and metastatic potential in OSCC. CD5L may influence signaling pathways involved in tumor progression, such as NF-κB, STAT3, or PI3K/AKT signaling [34], which are known to regulate cell proliferation, survival, and invasion in OSCC. Further studies will be required to clarify the downstream signaling pathways through which CD5L contributes to tumor progression.

Arecoline, the major alkaloid of areca nut, has been widely recognized as a key etiological factor in OSCC, particularly in regions where betel nut chewing is prevalent. An interesting finding of this study is that arecoline treatment increased CD5L expression in GNM cells. Previous studies have demonstrated that arecoline can induce oxidative stress, inflammation, and epithelial–mesenchymal transition (EMT), thereby promoting oral carcinogenesis [35]. Our findings suggest that CD5L expression may be upregulated in response to arecoline exposure, indicating a potential molecular link between betel nut chewing and OSCC progression. Our study may provide additional insights into the mechanisms underlying areca nut–related oral carcinogenesis [36,37].

Saliva has recently gained increasing attention as a valuable diagnostic biofluid for oral disease detection. Because OSCC develops in the oral cavity, tumor-derived molecules can be readily released into saliva, making salivary biomarkers particularly attractive for noninvasive cancer screening. In this study, CD5L was significantly elevated in the saliva of patients with late-stage OSCC. In contrast, immunohistochemical (IHC) staining was unable to significantly distinguish between early-stage and late-stage oral cancer. Although the discriminative performance of CD5L alone was modest, its significant association with late-stage oral cancer under clinically heterogeneous conditions suggests potential utility as an adjunctive biomarker for disease progression assessment. These findings highlight the potential of salivary CD5L as a non-invasive adjunctive biomarker for pathological staging, with promising clinical applications in disease monitoring and prognostic stratification.

Several limitations of this study should be acknowledged. Acquiring healthy control tissues and tissues from patients with early-stage disease was challenging, resulting in unequal sample sizes across groups for IHC quantitative analysis. This imbalance may have affected the statistical power and the robustness of group comparisons. The present study did not include dose–response analyses or comparative potency assessments between normal gingival and OSCC cells under arecoline stimulation. Therefore, whether arecoline-induced CD5L regulation exhibits OSCC-specific responsiveness remains undetermined. Future studies incorporating dose-dependent and comparative cellular analyses are warranted to further clarify the mechanistic and cell-type-specific regulation of CD5L.

## 5. Conclusions

In clinical practice, accurate pathological staging requires invasive tissue biopsy followed by histopathological evaluation. In contrast, our findings indicate that salivary CD5L can serve as a non-invasive adjunctive approach for assessing disease progression and biological aggressiveness in OSCC, although further validation is required before clinical application. This non-invasive approach may substantially facilitate early detection and timely intervention, while enhancing patient compliance and willingness to participate in screening programs.

## Figures and Tables

**Table 1:** Demographic and clinical characteristics (*n* = 116).

		Group			*p* Value	Phi/Cramer’sV
		Healthy Control (*n* = 36)	Early-Stage OSCC (*n* = 31)	Late-Stage OSCC (*n* = 49)		
Sex	Male	35	31	48		
	Female	1	0	1		
* Age (mean ± SD)		48.47 ± 18.01	58.89 ± 10.68	57.74 ± 9.99	0.003	
Alcohol	No	27	10	11	<0.001	0.47
	Yes	9 (25%)	21 (68%)	35 (71%)		
# Betel nut chewing	No	25	1	4	<0.001	0.679
	Yes	11 (30%)	30 (97%)	42 (86%)		
# Cigarette smoking	No	22	2	3	<0.001	0.614
	Yes	14 (38%)	29 (94%)	43 (88%)		
# Cancer status	Primary cancer		33 (58%)	51 (67%)	0.513	0.171
	Recurrent cancer		7 (13%)	11 (14%)		
	Metastatic cancer		2 (3%)	3 (4%)		
	Second primary cancer		6 (26%)	14 (12%)		
Tumor location	Missing data		0	1		
	Buccal mucosa		14 (45%)	14 (29%)		
	Gingiva		5 (16%)	15 (31%)		
	Lip		3 (10%)	2 (4%)		
	Tongue		6 (19%)	11 (22%)		
	Palate		1 (3%)	1 (2%)		
	Mouth floor		1 (3%)	2 (4%)		
	Retromolar trigone		1 (3%)	3 (6%)		

* Using ANOVA; # using Fisher’s exact test; all others were analyzed using the chi-square test.

**Table 2:** ELISA analysis of the CD5L expression in salivary specimens.

	Mean	S.D	S.E	95% CI Mean		Min	Max	Post Hoc Compared (Cohen’s d)	
				Lower	Upper			Healthy Control	Early- Stage
Healthy Control (*n* = 36)	173.94	91.82	15.30	142.87	205.01	40.27	372.61	-	-
Early-stage (*n* = 31)	181.44	124.27	22.32	135.86	227.02	15.97	593.42	0.96 (0.07)	-
Late-stage (*n* = 49)	345.96	303.54	43.36	258.77	433.14	22.44	1341.36	0.001 (0.77)	0.003 (0.70)

Welch ANOVA, post hoc: Games–Howell.

**Table 3:** Quantification of immunostaining intensity and area fraction in healthy and tumor tissues.

Group	Mean Intensity	Integrated Density	Area Fraction (%)
Healthy control (*n* = 5)	203.89 ± 2.43	12,601,174 ± 11,079,623	8.98 ± 7.33
Early-stage (*n* = 7)	183.30 ± 10.47	63,835,296 ± 32,366,842	48.33 ± 20.58
Late-stage (*n* = 22)	176.94 ± 19.03	72,058,214 ± 33,487,621	55.88 ± 20.42
*p* value	*p* < 0.001	*p* < 0.001	*p* < 0.001
Post Hoc			
Healthy vs. Early	*p* < 0.01 (2.49) ^a^	*p* < 0.001 (1.84) ^a^	*p* < 0.001 (2.46) ^a^
Healthy vs. Late	*p* < 0.001 (1.73) ^a^	*p* < 0.001 (2.05) ^a^	*p* < 0.001 (2.71) ^a^
Early vs. Late	*p* = 0.18 (0.4) ^a^	*p* = 0.42 (0.25) ^a^	*p* = 0.27 (0.36) ^a^

Welch ANOVA, post hoc: Games–Howell. ^a^ effect size (Cohen’s d). Data are presented as mean ± standard deviation (SD) from three independent replicates in each specimen. Healthy, early, and late represent the healthy control, early-stage, and late-stage groups, respectively.

## Data Availability

The datasets generated and/or analyzed during the current study are available from the corresponding authors on reasonable request. Due to privacy and ethical restrictions, the data are not publicly available.

## Supplementary Materials

The following supporting information can be downloaded at: https://www.mdpi.com/article/10.3390/diagnostics16121856/s1, Figure S1: Efficiency of CD5L knockdown by siRNA in GNM cells.

## References

[1] Cheng F.C., Wang L.H., Lin H.P., Chiang C.P. (2023). Morbidity and mortality of oral cancer in Taiwan: Trends from 2000 to 2021. J. Dent. Sci..

[2] Geum D.H., Roh Y.C., Yoon S.Y., Kim H.G., Lee J.H., Song J.M., Lee J.Y., Hwang D.S., Kim Y.D., Shin S.H. (2013). The impact factors on 5-year survival rate in patients operated with oral cancer. J. Korean Assoc. Oral Maxillofac. Surg..

[3] Yang Y.H. (2025). Oral cancer in Taiwan. Oral Dis..

[4] Tsai E., Walker B., Wu S.C. (2024). Can oral cancer screening reduce late-stage diagnosis, treatment delay and mortality? A population-based study in Taiwan. BMJ Open.

[5] Gonzalez-Ruiz I., Ramos-Garcia P., Ruiz-Avila I., Gonzalez-Moles M.A. (2023). Early Diagnosis of Oral Cancer: A Complex Polyhedral Problem with a Difficult Solution. Cancers.

[6] Ravindran S., Ranganathan S., R K., J N., A S., Kannan S.K., Prasad K D., Marri J., K R. (2025). The role of molecular biomarkers in the diagnosis, prognosis, and treatment stratification of oral squamous cell carcinoma: A comprehensive review. J. Liq. Biopsy.

[7] Brocklehurst P.R., Baker S.R., Speight P.M. (2009). Factors affecting the referral of potentially malignant lesions from primary dental care: A pilot study in South Yorkshire. Prim. Dent. Care.

[8] Kang Y.J., Park G., Park S.Y., Kim T., Kim E., Heo Y., Lee C., Jeong H.S. (2024). Extra-Capsular Spread of Lymph Node Metastasis in Oral, Oropharyngeal and Hypopharyngeal Cancer: A Comparative Subsite Analysis. Cancers.

[9] Hsu P.C., Huang J.H., Tsai C.C., Lin Y.H., Kuo C.Y. (2025). Early Molecular Diagnosis and Comprehensive Treatment of Oral Cancer. Curr. Issues Mol. Biol..

[10] Kumari S., Kumar N., Khan M.S., Sultana S., Khalid M. (2026). Early detection in oral cancer: Are we ready for AI-driven precision?. Ann. Med. Surg..

[11] Winck F.V., Prado Ribeiro A.C., Ramos Domingues R., Ling L.Y., Riano-Pachon D.M., Rivera C., Brandao T.B., Gouvea A.F., Santos-Silva A.R., Coletta R.D. (2015). Insights into immune responses in oral cancer through proteomic analysis of saliva and salivary extracellular vesicles. Sci. Rep..

[12] Bernhardt G.V., Bernhardt K.L., Pinto J.R.T., Vashe A. (2025). Metabolomics in cancer detection: A review of techniques, biomarkers, and clinical utility. Biomedicine.

[13] Chou C.W., Lin C.R., Chung Y.T., Tang C.S. (2023). Epidemiology of Oral Cancer in Taiwan: A Population-Based Cancer Registry Study. Cancers.

[14] Pandey R., Ngaju P., Janghorban M., Abuelazm H., Malaeb K., Aryal K.P., Mahato K., Chandra P. (2024). Noninvasive Biomarkers for Disease Diagnosis and Health Monitoring. Biosensors for Personalized Healthcare.

[15] Hu S., Arellano M., Boontheung P., Wang J., Zhou H., Jiang J., Elashoff D., Wei R., Loo J.A., Wong D.T. (2008). Salivary proteomics for oral cancer biomarker discovery. Clin. Cancer Res..

[16] Owecki W., Wojtowicz K., Nijakowski K. (2025). Salivary Extracellular Vesicles in Detection of Head and Neck Cancers: A Systematic Review. Int. J. Nanomed..

[17] Pedersen A.M.L., Sorensen C.E., Proctor G.B., Carpenter G.H., Ekstrom J. (2018). Salivary secretion in health and disease. J. Oral Rehabil..

[18] Goto T., Kishimoto T., Iwawaki Y., Fujimoto K., Ishida Y., Watanabe M., Nagao K., Ichikawa T. (2020). Reliability of Screening Methods to Diagnose Oral Dryness and Evaluate Saliva Secretion. Dent. J..

[19] Vila T., Rizk A.M., Sultan A.S., Jabra-Rizk M.A. (2019). The power of saliva: Antimicrobial and beyond. PLoS Pathog..

[20] Townsend S., Fanning L., O’Kennedy R. (2008). Salivary Analysis of Drugs—Potential and Difficulties. Anal. Lett..

[21] Karthikeyan P., Aswath N. (2016). Stress as an etiologic co-factor in recurrent aphthous ulcers and oral lichen planus. J. Oral. Sci..

[22] Shah B., Ashok L., Sujatha G.P. (2009). Evaluation of salivary cortisol and psychological factors in patients with oral lichen planus. Indian J. Dent. Res..

[23] Ohashi M., Iwase M., Nagumo M. (1999). Elevated production of salivary nitric oxide in oral mucosal diseases. J. Oral Pathol. Med..

[24] Rocha F.S., Jesus R.N., Rocha F.M., Moura C.C., Zanetta-Barbosa D. (2014). Saliva versus peri-implant inflammation: Quantification of IL-1beta in partially and totally edentulous patients. J. Oral. Implantol..

[25] Hoffmann R.R., Yurgel L.S., Campos M.M. (2011). Evaluation of salivary endothelin-1 levels in oral squamous cell carcinoma and oral leukoplakia. Regul. Pept..

[26] Wang W.C., Huang M.Y., Chen Y.K., Lan W.C., Shieh T.M., Shih Y.H. (2021). Salivary Exosome Proteomics and Bioinformatics Analysis in 7,12-Dimethylbenz[a]anthracene-Induced Oral Cancer with Radiation Therapy-A Syrian Golden Hamster Model. Diagnostics.

[27] Schindelin J., Arganda-Carreras I., Frise E., Kaynig V., Longair M., Pietzsch T., Preibisch S., Rueden C., Saalfeld S., Schmid B. (2012). Fiji: An open-source platform for biological-image analysis. Nat. Methods.

[28] Su M.J., Ho C.H., Yeh C.C. (2024). Association of alcohol consumption, betel nut chewing, and cigarette smoking with mortality in patients with head and neck cancer among the Taiwanese population: A nationwide population-based cohort study. Cancer Epidemiol..

[29] Chuang H.C., Tsai M.H., Lin Y.T., Chou M.H., Yang K.L., Chien C.Y. (2022). Systemic and Local Effects Among Patients With Betel Quid-Related Oral Cancer. Technol. Cancer Res. Treat..

[30] Sanchez-Moral L., Paul T., Martori C., Font-Diaz J., Sanjurjo L., Aran G., Tellez E., Blanco J., Carrillo J., Ito M. (2023). Macrophage CD5L is a target for cancer immunotherapy. EBioMedicine.

[31] Choi E.S., Faruque H.A., Kim J.H., Kim K.J., Choi J.E., Kim B.A., Kim B., Kim Y.J., Woo M.H., Park J.Y. (2021). CD5L as an Extracellular Vesicle-Derived Biomarker for Liquid Biopsy of Lung Cancer. Diagnostics.

[32] Aran G., Sanjurjo L., Barcena C., Simon-Coma M., Tellez E., Vazquez-Vitali M., Garrido M., Guerra L., Diaz E., Ojanguren I. (2018). CD5L is upregulated in hepatocellular carcinoma and promotes liver cancer cell proliferation and antiapoptotic responses by binding to HSPA5 (GRP78). FASEB J..

[33] Zhang X., Liu X., Zhu K., Zhang X., Li N., Sun T., Fan S., Dai L., Zhang J. (2022). CD5L-associated gene analyses highlight the dysregulations, prognostic effects, immune associations, and drug-sensitivity predicative potentials of LCAT and CDC20 in hepatocellular carcinoma. Cancer Cell. Int..

[34] LaFargue C.J., Amero P., Noh K., Mangala L.S., Wen Y., Bayraktar E., Umamaheswaran S., Stur E., Dasari S.K., Ivan C. (2023). Overcoming adaptive resistance to anti-VEGF therapy by targeting CD5L. Nat. Commun..

[35] Wang T.H., Shen Y.W., Chen H.Y., Chen C.C., Lin N.C., Shih Y.H., Hsia S.M., Chiu K.C., Shieh T.M. (2024). Arecoline Induces ROS Accumulation, Transcription of Proinflammatory Factors, and Expression of KRT6 in Oral Epithelial Cells. Biomedicines.

[36] Shieh T.M., Lin N.C., Shen Y.W., Lan W.C., Shih Y.H. (2025). Epithelium-derived exosomal dipeptidyl peptidase-4 involved in arecoline-induced oral submucous fibrosis. Biochim. Biophys. Acta Mol. Basis Dis..

[37] Tu H.F., Chen M.Y., Lai J.C., Chen Y.L., Wong Y.W., Yang C.C., Chen H.Y., Hsia S.M., Shih Y.H., Shieh T.M. (2019). Arecoline-regulated ataxia telangiectasia mutated expression level in oral cancer progression. Head Neck.

