# Supplementary figures and images for: Salivary CD5L as a Potential Non-Invasive Biomarker for Pathological Staging and Prognostic Assessment in Oral Squamous Cell Carcinoma
Source: Diagnostics (Basel). 2026 Jun 16;16(12):1856. doi: 10.3390/diagnostics16121856 (PMC13297783; doi:10.3390/diagnostics16121856)

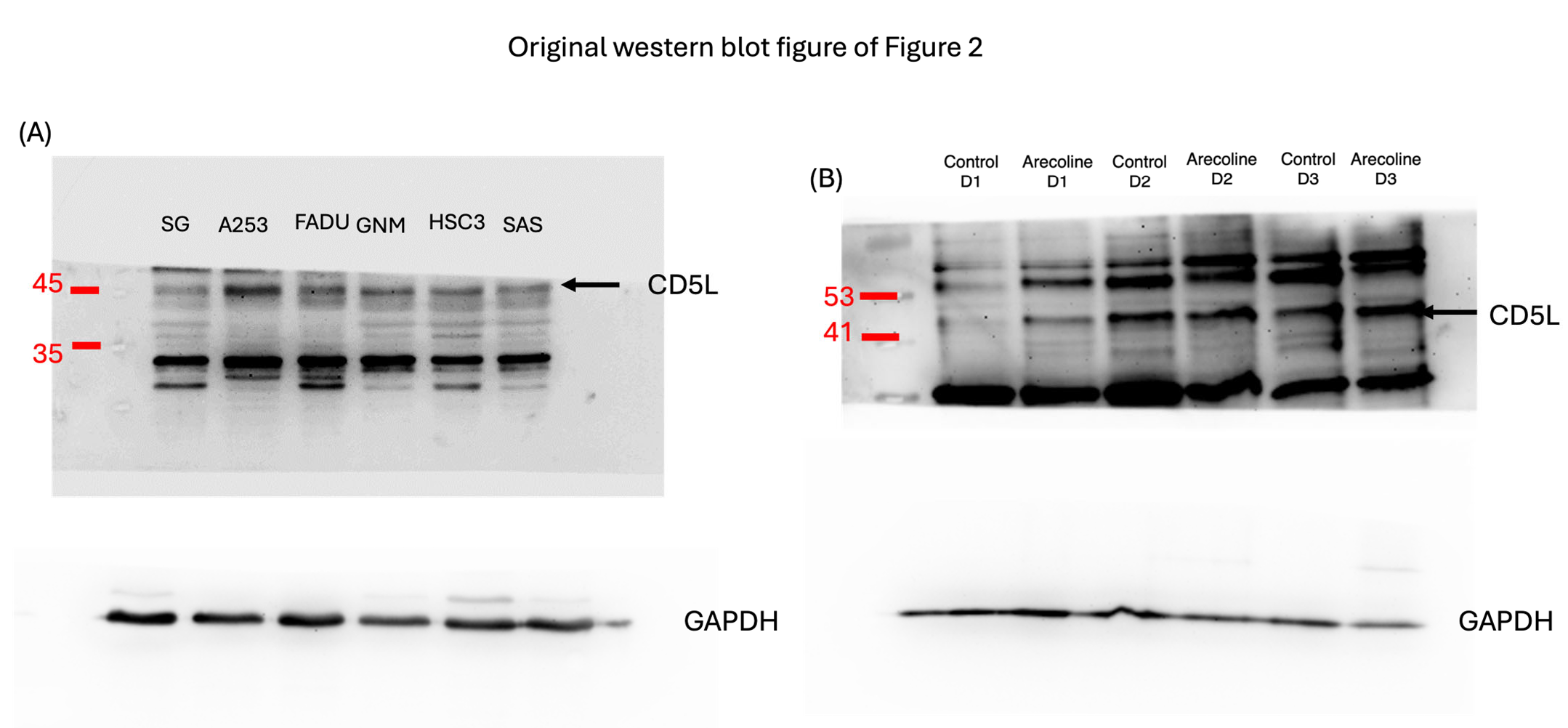

Supplement: Supplementary file 1 [file diagnostics-16-01856-s001.zip › diagnostics-4333449-supplementary.png]
